# Carbamazepine Increases the Risk of Sudden Cardiac Arrest by a Reduction of the Cardiac Sodium Current

**DOI:** 10.3389/fcell.2022.891996

**Published:** 2022-06-03

**Authors:** Lixia Jia, Talip E. Eroglu, Ronald Wilders, Arie O. Verkerk, Hanno L. Tan

**Affiliations:** ^1^ Department of Clinical and Experimental Cardiology, Heart Center, Amsterdam UMC, University of Amsterdam, Amsterdam, Netherlands; ^2^ Division of Pharmacoepidemiology and Clinical Pharmacology, Utrecht Institute for Pharmaceutical Sciences, Utrecht University, Utrecht, Netherlands; ^3^ Department of Cardiology, Herlev and Gentofte Hospital, University of Copenhagen, Gentofte, Denmark; ^4^ Department of Medical Biology, Amsterdam Cardiovascular Sciences, Amsterdam UMC, University of Amsterdam, Amsterdam, Netherlands; ^5^ Netherlands Heart Institute, Utrecht, Netherlands

**Keywords:** anti-epileptic drugs, sudden cardiac arrest, risk association, cardiomyocytes, sodium current, action potentials

## Abstract

**Aim:** To assess the risk of sudden cardiac arrest (SCA) associated with the use of carbamazepine (CBZ) and establish the possible underlying cellular electrophysiological mechanisms.

**Methods:** The SCA risk association with CBZ was studied in general population cohorts using a case–control design (*n* = 5,473 SCA cases, 21,866 non-SCA controls). Effects of 1–100 µM CBZ on action potentials (APs) and individual membrane currents were determined in isolated rabbit and human cardiomyocytes using the patch clamp technique.

**Results:** CBZ use was associated with increased risk of SCA compared with no use (adjusted odds ratio 1.90 [95% confidence interval: 1.12–3.24]). CBZ reduced the AP upstroke velocity of rabbit and human cardiomyocytes, without prominent changes in other AP parameters. The reduction occurred at ≥30 µM and was frequency-dependent with a more pronounced reduction at high stimulus frequencies. The cardiac sodium current (I_Na_) was reduced at ≥30 μM; this was accompanied by a hyperpolarizing shift in the voltage-dependency of inactivation. The recovery from inactivation was slower, which is consistent with the more pronounced AP upstroke velocity reduction at high stimulus frequencies. The main cardiac K^+^ and Ca^2+^ currents were unaffected, except reduction of L-type Ca^2+^ current by 100 µM CBZ.

**Conclusion:** CBZ use is associated with an increased risk of SCA in the general population. At concentrations of 30 µM and above, CBZ reduces AP upstroke velocity and I_Na_ in cardiomyocytes. Since the concentration of 30 µM is well within the therapeutic range (20–40 µM), we conclude that CBZ increases the risk of SCA by a reduction of the cardiac I_Na_.

## 1 Introduction

Sudden cardiac arrest (SCA) is a global public health problem with an annual incidence of 40–100 per 100,000 individuals (Fishman et al., 2010; Hayashi et al., 2015). SCA accounts for 50% of deaths from cardiovascular disease and 15–20% of all deaths in industrialized societies (Zipes and Wellens 1998; Wong et al., 2019). Most cases of SCA are caused by cardiac arrhythmias (ventricular fibrillation (VF) or ventricular tachycardia (VT)). Such arrhythmias may arise from functional changes in the ion channels that underlie the cardiac action potential (AP) (Antzelevitch and Burashnikov 2011). These functional changes may be evoked by various drugs used for the treatment of cardiac or non-cardiac conditions. This is best known for drugs that affect cardiac repolarization (QT prolonging drugs) (Haverkamp et al., 2000). However, there is increasing recognition that it also applies to drugs that affect cardiac depolarization (Bardai et al., 2013). An example of such drugs are anti-epileptic drugs (AEDs) (Bardai et al., 2015). Some AEDs are primarily developed for blocking neuronal ion channels, e.g., voltage-gated Na^+^, Ca^2+^ or K^+^ channels, while other AEDs act by impacting on neurotransmitters such as γ-aminobutyric acid (Davies 1995; Sills and Rogawski 2020). Importantly, neuronal and cardiac ion channel isoforms are highly homologous (Heinemann et al., 1994; Fozzard and Hanck 1996). Thus, AEDs may not only affect neuronal electrical activity but may also act on cardiac ion channels, thereby causing cardiac arrhythmias (Danielsson et al., 2005). Accordingly, the increased SCA risk of epilepsy patients may be partly explained by AED use (Bardai et al., 2015).

Carbamazepine (CBZ) is a prime example of such drugs, because it has high efficacy in the treatment of epilepsy (Pellock 2000) through various mechanism, including block of neuronal Na^+^ channels (Ragsdale and Avoli 1998; Catterall 1999; Sun et al., 2006; Lasoń et al., 2013). CBZ may also impact on cardiac electrophysiology as suggested by several CBZ-related case reports and retrospective studies, which report bradycardia, sinoatrial and atrioventricular block, QRS interval prolongation, cardiac arrhythmias, and cardiac arrest, as summarized in Table 1 (Beermann et al., 1975; Hamilton 1978; Herzberg 1978; Boesen et al., 1983; Leslie et al., 1983; Benassi et al., 1987; Kasarskis et al., 1992; Hojer et al., 1993; Schmidt and Schmitz-Buhl 1995; Koutsampasopoulos et al., 2014). Still, the underlying electrophysiological mechanism is not completely understood. Our current study has two aims: 1) to establish whether CBZ is associated with increased SCA risk in a large dataset from a cohort that was specifically designed to study SCA in the general population; 2) to establish the effects of CBZ on cardiac APs and individual membrane currents of rabbit and human cardiomyocytes using patch clamp methodology.

**TABLE 1:** Cardiac arrhythmias observed in patients using CBZ.

Source	Sex/Age (years) of Patient	Cardiac Arrhythmia Reported	CBZ Dose (Daily) or Serum/Plasma Level
Beermann et al. (1975)	F/66	3rd degree AV block	1,200 mg
Herzberg (1978)	F/85	sinus bradycardia	1,000 mg
Hamilton (1978)	F/77	sinus bradycardia	1,200 mg
Leslie et al. (1983)	M/50	sinus arrest	overdose (20 g), plasma level 62 mg/L (261 µM)
Boesen et al. (1983)	F/72	3rd degree AV block	400 mg
	F/82	SA block	600 mg
	F/86	SA block	400 mg
Benassi et al. (1987)	F/55	3rd degree AV block	800 mg, plasma level 8.5 μg/mL
	F/59	3rd degree AV block	800 mg, plasma level 4.7 μg/mL
Kasarskis et al. (1992)	F/58	bradycardia, AV block, sinus arrest	peak serum level 79.4 µM
Hojer et al. (1993)	M/34	ventricular fibrillation	peak serum level 218 µM
	M/54	AV block	peak serum level 285 µM
	M/83	3rd degree AV block	peak serum level 220 µM
	F/20	QRS widening	peak serum level 176 µM
Schmidt and Schmitz-Buhl (1995)	not reported	bradycardia/AV block (*n* = 2), cardiac arrest (*n* = 2)	overdose (dose not reported)
Koutsampasopoulos et al. (2014)	F/82	3rd degree AV block	1,200 mg

AV, atrioventricular; F, female; M, male; SA, sinoatrial.

## 2 Methods and Materials

### 2.1 Epidemiological Studies

We studied the SCA risk associated with CBZ use in a case–control design. Cases were patients who suffered out-of-hospital SCA with presumed cardiac causes in the Amsterdam Resuscitation Studies (ARREST) registry. ARREST is an ongoing, prospective, population-based registry that we designed to study the occurrence and outcome of out-of-hospital SCA in the general population. Patients are collected in collaboration with dispatch centers, ambulance personnel, pharmacies and hospitals in one contiguous study region in the Netherlands (2.6 million inhabitants, urban and rural areas), thereby assuring collection of >95% of all out-of-hospital SCA patients in the study region and minimizing inclusion bias (Blom et al., 2014). Each out-of-hospital SCA case was matched with up to five non-SCA controls based on age, sex and index-date (SCA-date). Non-SCA controls were randomly drawn from the general population using the PHARMO Database Network (Kuiper et al., 2020), which contains, among other things, complete medication data from the community pharmacists across the Netherlands.

Drug dispensing records for drugs prescription were obtained from computerized databases of pharmacists. Use of CBZ was defined as having a drug-dispensing record within 90 days prior to index-date. We chose a period of 90 days, since, in the Netherlands, prescription length for drugs used for chronic disease is 90 days.

For all cases and controls, we included cardiovascular disease and diabetes mellitus in our analyses because these are known risk factors for SCA. We derived cardiovascular disease and diabetes mellitus by using medication use as proxies as we did previously (Eroglu et al., 2020). Cardiovascular disease was defined by use of β-adrenoceptor blockers, calcium channel blockers, diuretics, renin-angiotensin system inhibitors, diuretics, antithrombotics, nitrates and statins. Diabetes mellitus was defined by use of antidiabetics. Patients were considered users of cardiovascular drugs and antidiabetics if there was any drug-dispensing record within 6 months prior to index-date.

### 2.2 Cellular Electrophysiological Studies

#### 2.2.1 Cell Preparations

Full details of rabbit ventricular and human atrial cell isolation procedures are provided in the Supplementary Material. The investigation using rabbits conformed to the Guide for the Care and Use of Laboratory Animals (NIH Publication 85–23, 1996) and was approved by the institutional animal experiments committee. The human atrial cardiomyocytes were isolated from explanted hearts of male patients with end-stage heart failure caused by ischemic cardiomyopathy. All patients were in New York Heart Association functional class IV and received standard therapy for chronic heart failure (Supplementary Table S1). Informed consent was obtained before heart transplantation, and the protocol complied with institutional guidelines.

#### 2.2.2 Action Potentials

**FIGURE 1:**
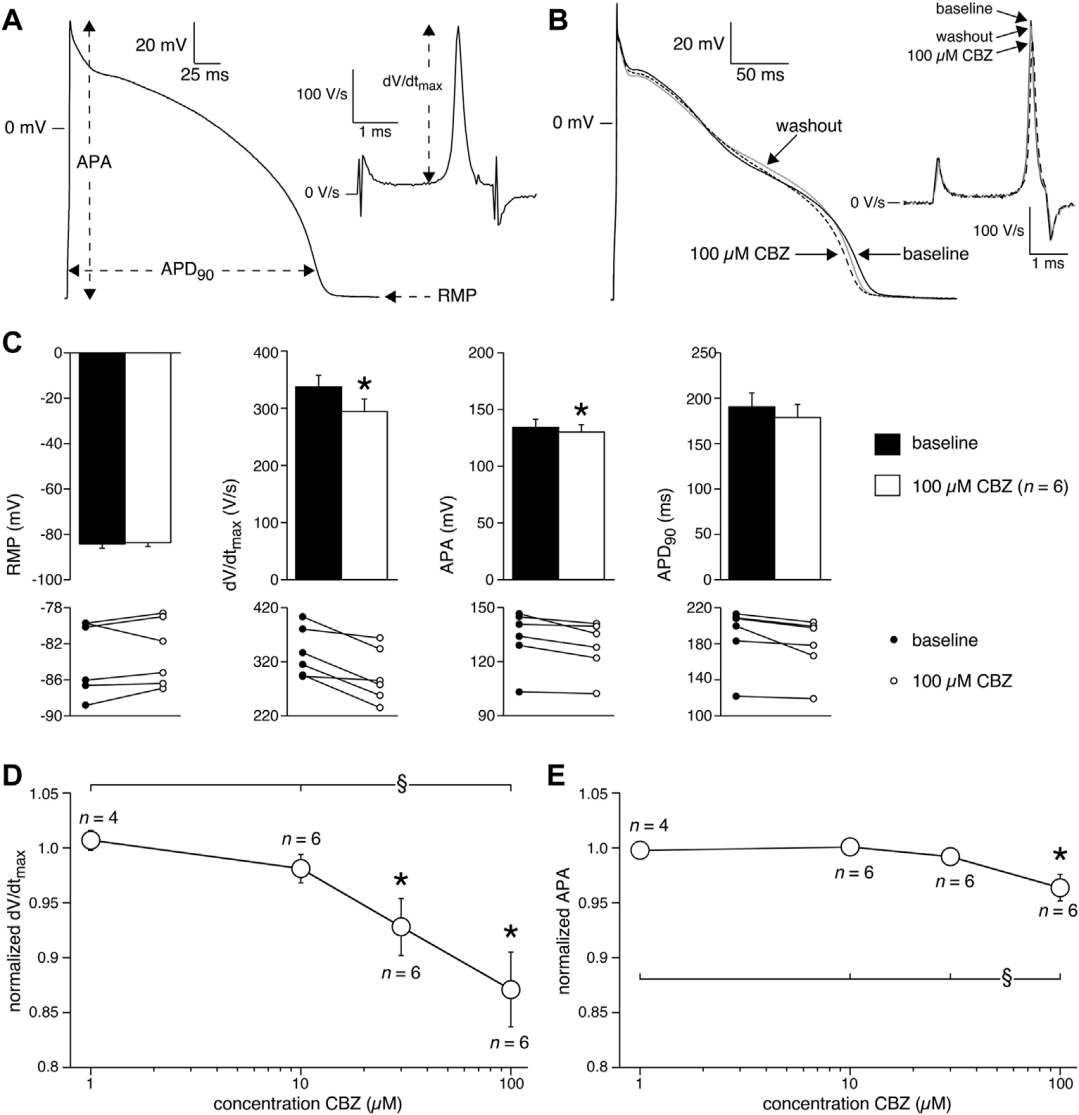
Carbamazepine (CBZ) reduces the action potential (AP) upstroke velocity and AP amplitude of rabbit ventricular cardiomyocytes. **(A)** AP recording illustrating the analyzed AP parameters. Inset: time derivative (dV/dt) of the AP upstroke on an expanded time scale. RMP, resting membrane potential; APA, AP amplitude; APD_90_, AP duration at 90% of repolarization; dV/dt_max_, maximal AP upstroke velocity. **(B)** Superimposed representative APs at 1 Hz under baseline conditions, in presence of 100 µM CBZ, and upon washout of the drug. Inset: time derivatives of the AP upstrokes. **(C)** Average AP characteristics at 1 Hz (top panels) and individual (paired) data points (bottom panels). *p < 0.05 CBZ versus baseline (One-Way RM ANOVA). **(D,E)** Average normalized dV/dt_max_ **(D)** and APA **(E)** at 1 Hz in response to 1, 10, 30, and 100 µM CBZ. Values are normalized to the values measured under baseline conditions. Numbers near symbols indicate the number of cells (*n*) measured at a given concentration. *p < 0.05 CBZ versus baseline (One-Way RM ANOVA); ^§^ *p* < 0.05 CBZ 100 µM versus lower concentrations (One-Way ANOVA).

APs were measured at 36 ± 0.2°C in modified Tyrode’s solution containing (in mM): NaCl 140, KCl 5.4, CaCl_2_ 1.8, MgCl_2_ 1.0, glucose 5.5, HEPES 5.0; pH 7.4 (NaOH). Patch pipettes were filled with solution composed of (in mM): K-gluconate 125, KCl 20, NaCl 5.0, K_2_ATP 2.0, HEPES 10; pH 7.2 (KOH). Detailed recording procedures are provided in the Supplementary Material. APs were evoked at stimulation rates of 0.2–4 Hz using square 3-ms current pulses through the patch pipette. To reduce variability in the moment of AP upstroke, stimulus amplitude was chosen such that the AP upstroke originated just before the end of the stimulus, as we described previously (Remme et al., 2006). The maximal AP upstroke velocity (dV/dt_max_) was determined from the first derivative of the AP upstroke from which the approximately constant initial dV/dt in response to the stimulus pulse was subtracted (Figure 1A, inset). In addition, we analyzed resting membrane potential (RMP), AP amplitude (APA), and AP duration at 90% repolarization (APD_90_), as also shown in Figure 1A. AP parameters from 10 consecutive APs were averaged.

#### 2.2.3 Membrane Current Measurements

The L-type Ca^2+^ current (I_Ca,L_), inward rectifier K^+^ current (I_K1_), delayed rectifier K^+^ current (I_K_), and transient outward K^+^ current (I_to1_) were all measured at 36 ± 0.2°C with the same solutions as used for the AP measurements. However, I_to1_ was measured in the presence of CdCl_2_ (0.25 mM) to block I_Na_ and I_Ca,L_, thereby also preventing activation of the outward Ca^2+^-activated Cl^−^ current (Verkerk et al., 2011). Suppression of these inward and outward currents allows accurate determination of I_to1_. The whole-cell sodium current (I_Na_) in freshly isolated cardiomyocytes is an extremely large and fast activating and inactivating membrane current, which for technical reasons cannot be reliably measured at a close-to-physiological temperature and normal Na^+^ gradients over the cell membrane (see Berecki et al. (2010) and primary references cited therein). Therefore, we measured I_Na_ at room temperature with modified bath and pipette solutions (including an identical Na^+^ concentration in pipette and bath solution), which allowed specific measurements of Na^+^ currents only. Bath solution for I_Na_ measurements contained (in mM): NaCl 7.0, CsCl 133, CaCl_2_ 1.8, MgCl_2_ 1.2, glucose 11.0, HEPES 5.0, and nifedipine 0.05; pH 7.4 (CsOH). Patch pipettes for I_Na_ measurements were filled with (in mM): NaCl 3.0, CsCl 133, MgCl_2_ 2.0, Na_2_ATP 2.0, TEA-Cl 2.0, EGTA 10, HEPES 5.0; pH 7.3 (CsOH). The membrane currents were measured with specific voltage clamp protocols as depicted in the insets to Figures 3–5 and described in detail in the Supplementary Material. Recording procedures and data analysis are also described in detail in the Supplementary Material.

#### 2.2.4 Preparation of Carbamazepine

CBZ obtained from Sigma-Aldrich (St. Louis, MO, US) was freshly dissolved every day in dimethyl sulfoxide (DMSO) as 100 mM stock and diluted in the bath solution to the desired concentration just before use. APs and membrane currents were measured in the presence of the vehicle DMSO and after wash-in of CBZ (1, 10, 30, or 100 µM) in the same cardiomyocytes. In order to obtain steady-state conditions, signals were recorded after a 5 min stimulation period, i.e. under baseline conditions, and 5 min after application of CBZ.

### 2.3 Statistics

Data are presented as mean ± SEM. The association between CBZ and SCA was estimated by calculating the adjusted odds ratio with 95% confidence interval using conditional logistic regression by adjusting for the use of cardiovascular drugs and antidiabetics. For the patch-clamp study, comparisons were made using One-Way ANOVA, One-Way Repeated Measures (RM) ANOVA, or Two-Way RM ANOVA, followed by pairwise comparison using the Student-Newman-Keuls *post hoc* test. For the epidemiological study, differences in baseline values for binary variables between cases and controls were tested using a chi-square test. Differences in baseline values for continuous variables between cases and controls were tested using an independent *t*-test. *p* < 0.05 defined statistical significance.

## 3 Results

### 3.1 Carbamazepine Use and the Risk of Sudden Cardiac Arrest

We first conducted a systematic study to establish whether CBZ use is associated with increased risk of SCA in the general population. We identified 5,473 SCA cases, and matched them to 21,866 non-SCA controls. The mean age of the cases was 68.8 years and 69.9% were male. As expected, the prevalence of cardiovascular drugs and antidiabetics was higher among the cases than controls (Table 2). We observed that the proportion of CBZ users was significantly higher among cases (*n* = 24, 0.44%) than among controls (*n* = 41, 0.19%) (Table 3). After adjusting for cardiovascular drugs and antidiabetics, we found that use of CBZ was associated with increased risk of SCA compared with no use of CBZ, with an adjusted odds ratio of 1.90 (95% confidence interval: 1.12–3.24; Table 3).

**TABLE 2:** Characteristics of cases and controls.

	Cases (*n* = 5,473)	Controls (*n* = 21,866)
Age, years (mean ± SD)	68.8 ± 14.0	68.8 ± 14.0
Male sex	3,823 (69.9%)	15,263 (69.8%)
Cardiovascular pharmacotherapy^a^		
Beta blockers	1,998 (36.5%)	3,839 (17.6%)
Digoxin	295 (5.4%)	334 (1.5%)
Renin-angiotensin system inhibitors	2,073 (37.9%)	4,802 (22.0%)
Calcium channel blockers	902 (16.5%)	2,016 (9.2%)
Antithrombotics	2,299 (42.0%)	4,853 (22.2%)
Diuretics	1,590 (29.1%)	2,712 (12.4%)
Nitrates	574 (10.5%)	841 (3.9%)
Antiarrhythmic drugs class 1 or 3^b^	114 (2.1%)	183 (0.8%)
Antidiabetics	936 (17.1%)	2,145 (9.8%)

a Defined as use within 6 months before index date.

b Defined as use within 90 days before index date.

**TABLE 3:** Carbamazepine (CBZ) and risk of out-of-hospital cardiac arrest.

	Cases (*n* = 5,473)	Controls (*n* = 21,866)	Crude Odds Ratio	Adjusted Odds Ratio
No use of CBZ	5,438 (99.4%)	21,807 (99.7%)	1.0 (reference)	1.0 (reference)
Use of CBZ	24 (0.44%)^a^	41 (0.19%)^a^	2.34 (1.42–3.89^b^)	1.90 (1.12–3.24^b^)

a Not included are 11 cases (0.20%) and 18 control (0.08%) who used CBZ in combination with other antiepileptic drugs.

b 95% confidence interval.

### 3.2 Effects of Carbamazepine on Action Potentials of Rabbit Ventricular Cardiomyocytes

Next, we characterized the effects of 1, 10, 30, and 100 µM CBZ on APs elicited at 1 Hz in rabbit ventricular cardiomyocytes. Figure 1B shows typical APs under baseline conditions (solid line), in the presence of 100 µM CBZ (dashed line), and upon washout of the drug (gray line). Exposure to 100 µM CBZ resulted in substantial alterations in AP morphology in comparison to baseline conditions, particularly a decrease in dV/dt_max_ and APA (as measures of cardiac depolarization) and a slight decrease of APD_90_ (as a measure of cardiac repolarization). The effects were partially reversible upon washout of the drug. Average data are shown in the top panels of Figure 1C, with the individual (paired) data of the 6 cells tested shown in the bottom panels. These data indicate that dV/dt_max_ and APA were significantly decreased by 12.9 ± 3.3% (294 ± 22 (CBZ) vs. 337 ± 20 V/s (baseline)) and 3.6 ± 1.2% (128 ± 6.5 (CBZ) vs. 133 ± 7.2 mV (baseline)), respectively. The effects of CBZ on dV/dt_max_ and APA were concentration dependent (Figures 1D,E). At 100 µM CBZ, RMP was unaffected (–83.0 ± 1.7 (CBZ) vs. –83.5 ± 1.8 mV (baseline)) and the small effect on APD_90_ (177 ± 14 (CBZ) vs. 189 ± 15 ms (baseline)) did not reach the level of statistical significance (Figure 1C). Similarly, no statistically significant effects on RMP and APD_90_ were observed at other stimulus frequencies or at lower CBZ concentrations (Supplementary Figure S1).

**FIGURE 2:**
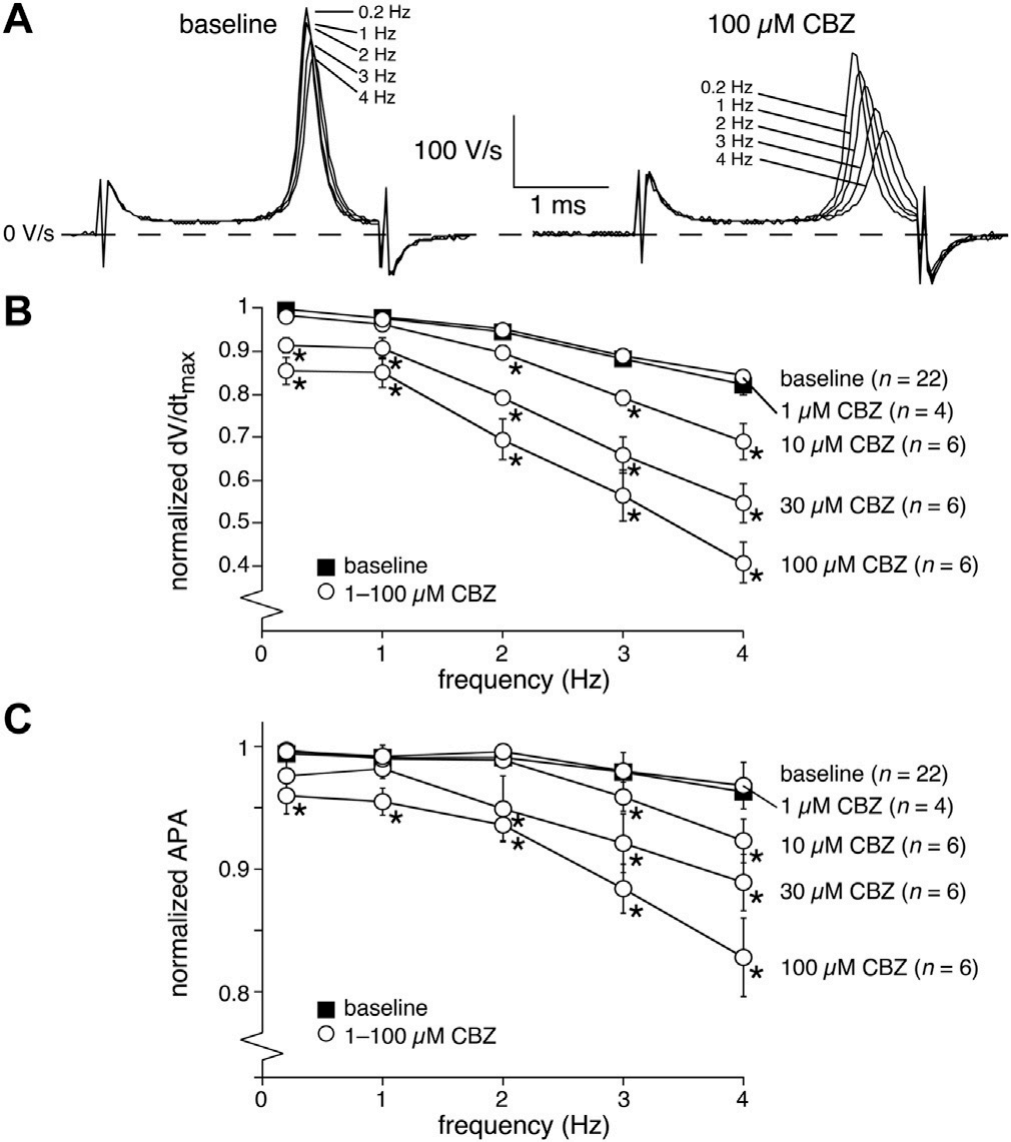
Carbamazepine (CBZ) reduces the AP upstroke velocity and amplitude of rabbit ventricular cardiomyocytes in a frequency dependent manner. **(A)** Superimposed typical time derivatives (dV/dt) during the AP upstroke phase at stimulus frequencies ranging from 0.2 to 4 Hz under baseline conditions (left) and in presence of 100 µM CBZ (right). **(B)** Average dV/dt_max_ under baseline conditions (filled squares) and in response to 1–100 µM CBZ (open circles) at stimulus frequencies ranging from 0.2 to 4 Hz. Values are normalized to the highest dV/dt_max_ measured at 0.2–4 Hz under baseline conditions. *p < 0.05 CBZ versus baseline (Two-Way RM ANOVA). See Supplementary Figure S2 for statistical significance of the frequency dependent effects. **(C)** Average APA under baseline conditions (filled squares) and in response to 1–100 µM CBZ (open circles) at stimulus frequencies ranging from 0.2 to 4 Hz. Values are normalized to the highest APA measured at 0.2–4 Hz under baseline conditions. *p < 0.05 CBZ versus baseline (Two-Way RM ANOVA).

The upstroke of APs in working cardiomyocytes is mainly due to I_Na_ (see Berecki et al. (2010) and primary references cited therein), which suggests that the CBZ-induced decrease in dV/dt_max_ is due to blockade of I_Na_. It is well-known that drugs may block I_Na_ in a voltage- and use-dependent manner (Bagal et al., 2015). The latter means that the amount of block may increase upon higher stimulus frequencies. Figure 2A shows typical AP time derivatives under baseline and 100 µM CBZ conditions at stimulus frequencies ranging from 0.2 to 4 Hz, while Figure 2B summarizes the average effects on dV/dt_max_ at 100 µM CBZ as well as lower concentrations. An increase in stimulus frequency resulted in a significantly lower dV/dt_max_ at every concentration tested (Figure 2B, filled squares; see also Supplementary Figure S2), consistent with a reduced I_Na_ recovery from inactivation at fast pacing rates (Berecki et al., 2010). In addition, the CBZ-induced decrease in dV/dt_max_ is more pronounced at higher stimulus frequencies (Figure 2B, open circles). For example, 100 µM CBZ decreased dV/dt_max_ by 14.1 ± 3.4% (294 ± 20 (CBZ) vs. 342 ± 18 V/s (baseline)) at 0.2 Hz, but by as much as 41.5 ± 12% (143 ± 21 (CBZ) vs. 264 ± 33 V/s (baseline)) at 4 Hz. Because APA and dV/dt_max_ are both importantly determined by I_Na_ (Krishnan and Antzelevitch 1991; Berecki et al., 2010), it is not surprising that the APA shows a largely similar concentration and frequency dependency as dV/dt_max_ (Figures 2B,C).

### 3.3 Effects of Carbamazepine on Membrane Currents of Rabbit Ventricular Cardiomyocytes

**FIGURE 3:**
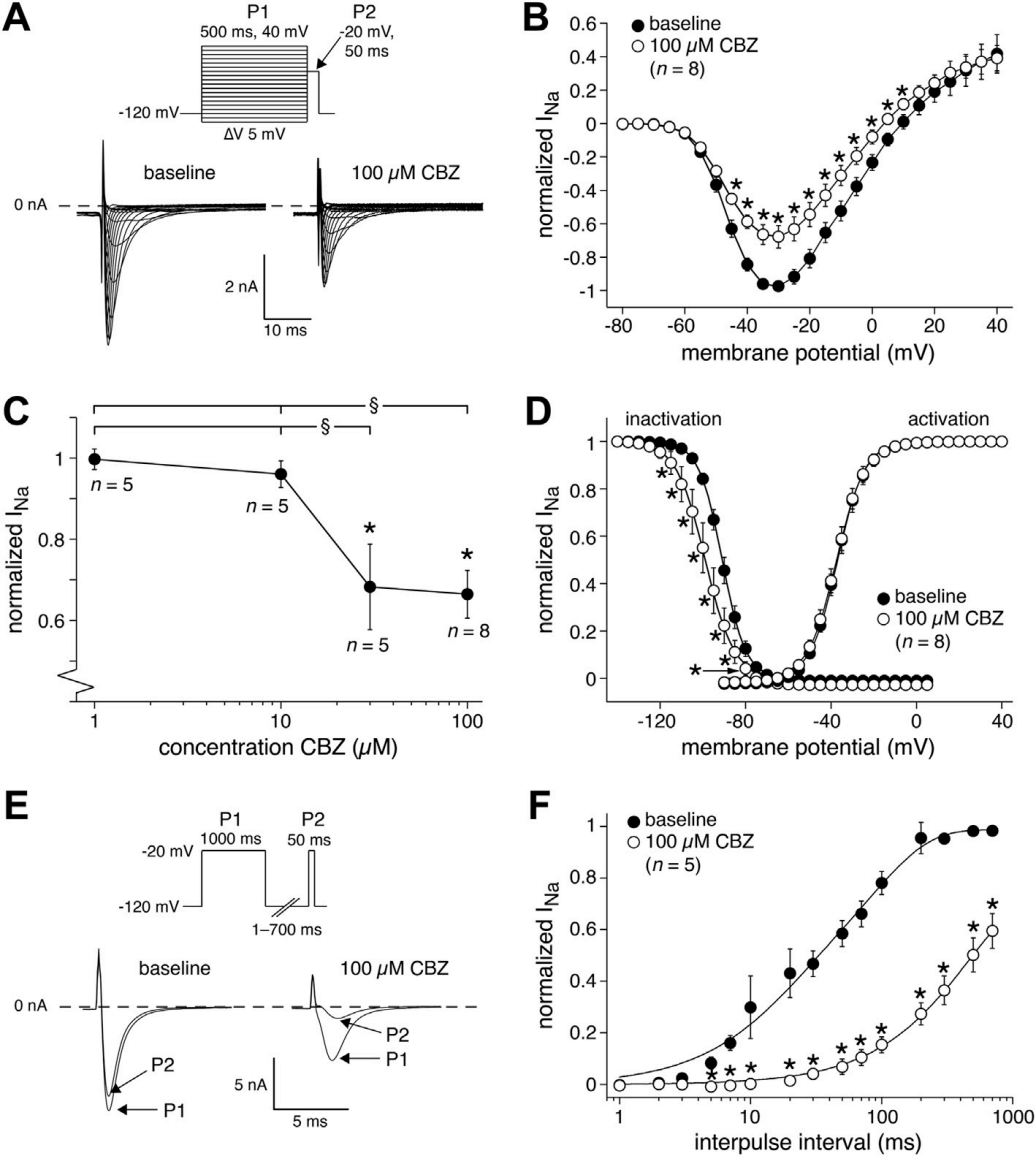
Carbamazepine (CBZ) reduces the sodium current (I_Na_) of rabbit ventricular cardiomyocytes in a use-dependent manner. **(A)** Typical I_Na_ recordings between −80 and 0 mV under baseline conditions and in presence of 100 µM CBZ. Inset: double-pulse voltage clamp protocol used to measure current-voltage (I–V) relationships **(B)** as well as the voltage dependency of (in)activation **(D)**. Cycle length was 5 s. **(B)** Average I-V relationship of I_Na_ under baseline conditions and in presence of 100 µM CBZ. I_Na_ was normalized to the maximal peak amplitude under baseline conditions, but peak current was set to −1 to retain the well-known inward direction of I_Na_. *p < 0.05 CBZ versus baseline (Two-Way RM ANOVA). **(C)** Concentration dependency of the CBZ effects on I_Na_ amplitude at −35 mV *p < 0.05 CBZ versus baseline (One-Way RM ANOVA); ^§^ *p* < 0.05 higher versus lower CBZ concentrations (One-Way ANOVA). **(D)** Voltage dependency of (in)activation. Solid lines are Boltzmann fits to the average data. *p < 0.05 CBZ versus baseline (Two-Way RM ANOVA). **(E,F)** Recovery from I_Na_ inactivation measured with a double-pulse protocol (E, inset). **(E)** Typical I_Na_ recordings under baseline conditions and in presence of 100 µM CBZ with an interpulse interval of 100 ms. **(F)** Average data. Solid lines are double-exponential fits to the average data. *p < 0.05 CBZ versus baseline (Two-Way RM ANOVA).

We next studied the effects of CBZ on the main membrane currents underlying cardiac APs in rabbit ventricular cardiomyocytes. First, we focused on the main current underlying the AP depolarization, i.e., I_Na_. Figure 3A shows typical I_Na_ recordings (at −80 to 0 mV) and Figure 3B shows the average current-voltage (I-V) relationships of I_Na_ under baseline conditions and in the presence of 100 µM CBZ. CBZ significantly decreased I_Na_ in the voltage range from −45 to +10 mV, e.g., by 30.3 ± 6.7% at −30 mV (67.8 ± 6.7% (CBZ) vs. 97.3 ± 2.0% (baseline) of the maximal peak amplitude under baseline conditions). Figure 3C shows the dose-dependency of the CBZ effects on I_Na_ and demonstrates that I_Na_ was also significantly reduced by 30 µM CBZ. Figure 3D shows the steady-state activation and inactivation curves for I_Na_ under baseline conditions and in the presence of 100 µM CBZ. While CBZ did not affect the voltage dependency of activation, the voltage dependency of inactivation was significantly shifted to more negative membrane potentials. On average, the negative shift in V_1/2_ was 6.2 ± 1.3 mV (−90.4 ± 1.8 (CBZ) vs. −84.3 ± 1.0 mV (baseline)), while the slope of the inactivation curve was not significantly different between baseline (−5.0 ± 0.9 mV) and CBZ (−5.4 ± 0.7 mV). Figures 3E,F, show the recovery from inactivation of I_Na_, with in Figure 3E typical I_Na_ recordings (bottom) obtained in response to a double-pulse protocol (top) with an interpulse interval of 100 ms, and in Figure 3F the average data with all interpulse intervals tested. CBZ results in a severe delay in the recovery from inactivation. For example, with an interpulse interval of 100 ms, recovery from inactivation was as large as 78.1 ± 4.5% at baseline, but only 15.4 ± 3.1% in the presence of CBZ.

**FIGURE 4:**
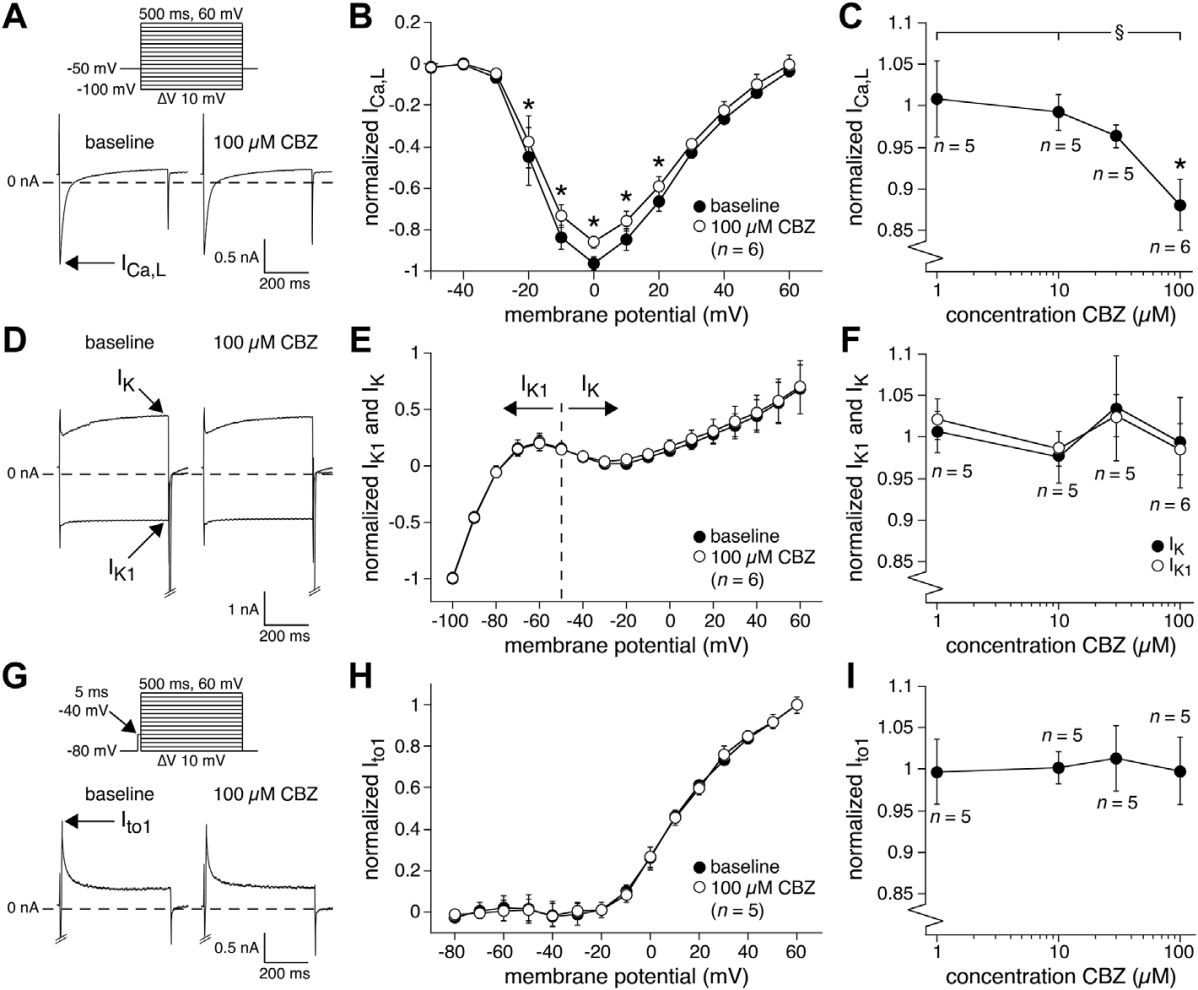
Carbamazepine (CBZ) reduces the L-type Ca^2+^ current of rabbit ventricular cardiomyocytes without affecting K^+^ currents. **(A)** Typical recordings of the L-type Ca^2+^ current (I_Ca,L_) at 0 mV under baseline conditions and in presence of 100 µM CBZ. Inset: voltage clamp protocol used. Cycle length was 2 s. **(B)** Average I-V relationship of I_Ca,L_ under baseline conditions and in presence of 100 µM CBZ. I_Ca,L_ was normalized to the maximal peak amplitude under baseline conditions, but peak current was set to −1 to retain the well-known inward direction of I_Ca,L_. *p < 0.05 CBZ versus baseline (Two-Way RM ANOVA). **(C)** Concentration dependency of the CBZ effect on I_Ca,L_ amplitude measured at 0 mV. *p < 0.05 CBZ versus baseline (One-Way RM ANOVA); ^§^ *p* < 0.05 CBZ 100 µM versus lower concentrations (One-Way ANOVA). **(D)** Typical recordings of the delayed rectifier K^+^ current (I_K_; at +60 mV) and inward rectifier K^+^ current (I_K1_; at −100 mV) under baseline conditions and in presence of 100 µM CBZ. Voltage clamp protocol as in panel **(A). (E)** Average I-V relationships of I_K_ and I_K1_ under baseline conditions and in presence of 100 µM CBZ. The currents were normalized to the current measured at −100 mV (and set to −1) under baseline conditions. **(F)** Concentration dependency of the CBZ effect on I_K1_ and I_K_ amplitude measured at −100 mV and +60 mV, respectively. **(G)** Typical recordings of the transient outward K^+^ current (I_to1_) at +60 mV under baseline conditions and in presence of 100 µM CBZ. Inset: voltage clamp protocol used. Cycle length was 5 s. **(H)** Average I-V relationships of I_to1_ under baseline conditions and in presence of 100 µM CBZ. I_to1_ was normalized to the current at +60 mV under baseline conditions. **(I)** Concentration dependency of the CBZ effect on I_to1_ amplitude at +60 mV.

Second, we studied the main currents underlying the AP repolarization. Although APD_90_ was not significantly affected by CBZ, a potential increase (or decrease) in outward currents can be balanced by a similar increase (or decrease) in inward currents, or vice versa. Figure 4A shows typical recordings (at 0 mV) and Figure 4B shows the average I-V relationships of the inward I_Ca,L_ under baseline conditions and in the presence of 100 µM CBZ. CBZ significantly decreased the I_Ca,L_ density in the voltage range from −20 to +20 mV (Figure 4B). Figure 4C shows that I_Ca,L_ was only significantly reduced at the highest concentration of CBZ tested, i.e., 100 µM. The reduction in peak I_Ca,L_ at 0 mV was 10.3 ± 3.7% (86.0 ± 3.0% (CBZ) vs. 96.2 ± 3.2% (baseline) of the maximal peak amplitude under baseline conditions). Figure 4D shows typical recordings and Figure 4E shows the average I-V relationships of the steady-state outward K^+^ currents, I_K_ and I_K1_, under baseline conditions and in the presence of 100 µM CBZ. Figure 4F shows the concentration dependency of I_K_ and I_K1_. Neither I_K_ nor I_K1_ were significantly affected by CBZ. Figure 4G shows typical recordings and Figure 4H shows the average I-V relationships of I_to1_ under baseline and 100 µM CBZ conditions. Figure 4I shows the concentration dependency of I_to1_. We observed no significant changes in the amplitude of I_to1_ at any voltage and concentration tested.

### 3.4 Effects of Carbamazepine on Action Potentials of Human Atrial Cardiomyocytes

**FIGURE 5:**
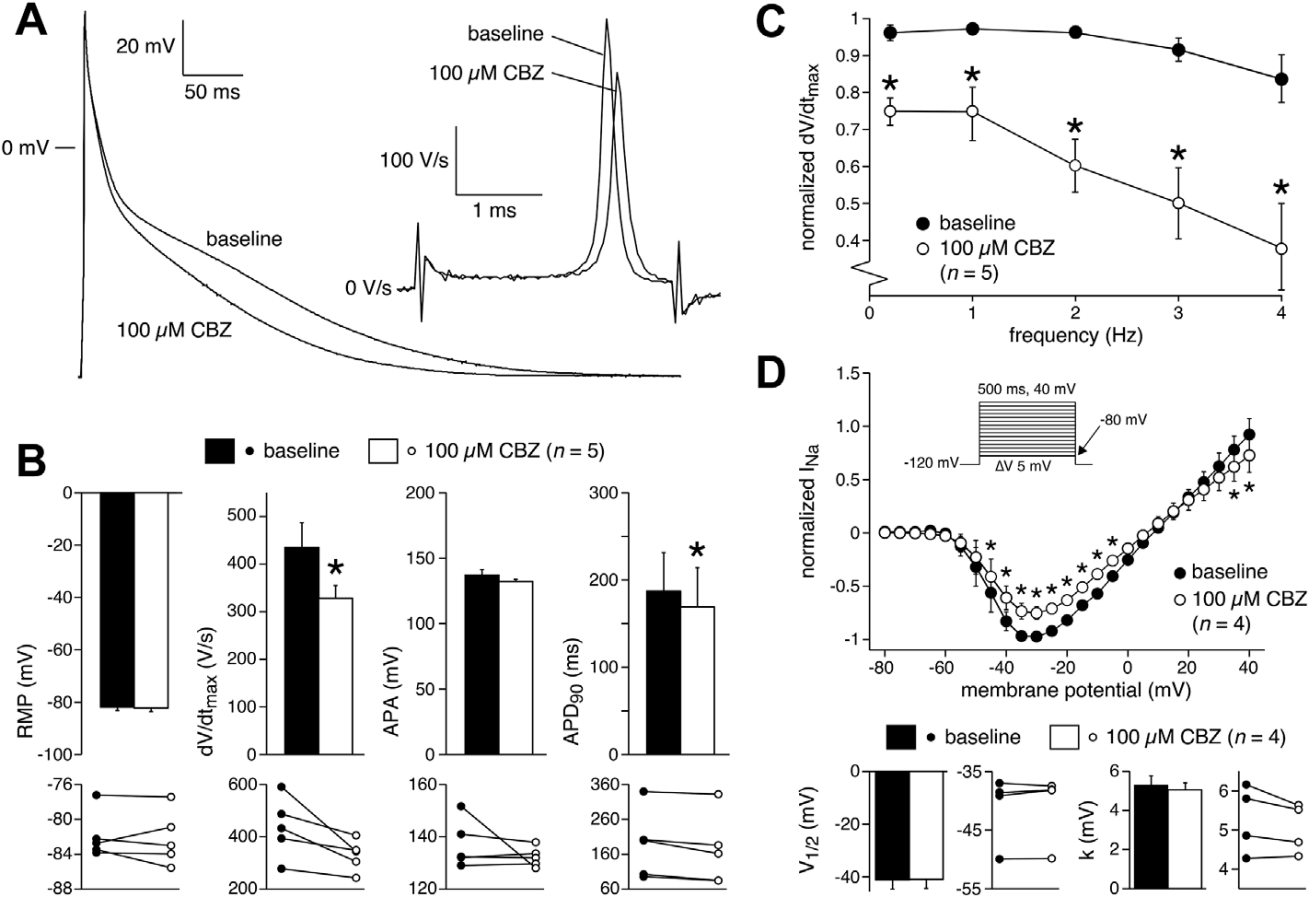
Carbamazepine (CBZ) affects human atrial electrophysiology. **(A)** Superimposed representative human atrial APs at a stimulus frequency of 1 Hz in control conditions and in presence of 100 µM CBZ. Inset: time derivatives during the AP upstroke phase. **(B)** Average AP characteristics at a stimulus frequency of 1 Hz under baseline conditions and in presence of 100 µM CBZ (top panels) and individual (paired) data points (bottom panels). *p < 0.05 CBZ versus baseline (One-Way RM ANOVA). **(C)** Average dV/dt_max_ in response to 100 µM CBZ at stimulus frequencies ranging from 0.2 to 4 Hz. Values are normalized to the highest dV/dt_max_ measured under baseline conditions. *p < 0.05 CBZ versus baseline (Two-Way RM ANOVA). **(D)** Average I-V relationship of I_Na_ under baseline conditions and in presence of 100 µM CBZ (top panel) and V_1/2_ and k of activation (bottom panels). Inset: voltage clamp protocol used. Cycle length was 5 s. I_Na_ was normalized to the maximal peak amplitude under baseline conditions, but peak current was set to −1 to retain the well-known inward direction of I_Na_. *p < 0.05 CBZ versus baseline (Two-Way RM ANOVA).

Having established the effects of CBZ on AP properties and membrane current of rabbit cardiomyocytes, we measured the effects of 100 µM CBZ on APs and I_Na_ density of freshly isolated human atrial cardiomyocytes to study whether these effects may also occur in the human heart. In patch clamp experiments on single isolated human atrial cardiomyocytes, the amount of quiescent, Ca^2+^-tolerant cells is typically low and non-depolarized cells are scarce (Verkerk et al., 2021). Here, we selected cardiomyocytes with an RMP of −75 mV or more negative, which generated stable APs after an initial 8–10 min period of continuous pacing at 1 Hz. Figure 5A shows typical APs at 1 Hz under baseline conditions and in the presence of 100 µM CBZ. Average AP parameters are summarized in the top panels of Figure 5B, with the individual (paired) data of the 5 cells tested shown in the bottom panels. Under baseline conditions, the pre-selected human atrial cardiomyocytes had an RMP of −81.9 ± 1.3 mV and a high maximum AP upstroke velocity, and the APs largely overshot the zero potential value. CBZ (100 µM) significantly reduced the AP upstroke velocity and significantly shortened AP duration, without affecting RMP or APA (Figure 5B). These effects are largely comparable to those in rabbit ventricular cardiomyocytes. For example, the AP upstroke velocity decreased significantly by 23.4 ± 6.5% (from 435 ± 58 (baseline) to 328 ± 30 V/s (CBZ)), while the APD_90_ was significantly decreased by 11.8 ± 3.5% (from 187 ± 49 (control) to 169 ± 51 ms (CBZ)). Furthermore, human APs showed a frequency dependency in maximum AP upstroke velocity with a decrease at higher frequencies (Figure 5C, filled circles). The frequency dependency in the presence of CBZ was more pronounced, indicating a similar use-dependent reduction of I_Na_ by CBZ (Figure 5C, open circles) as found in rabbit cardiomyocytes. Figure 5D (top panel), shows the I-V relationships of I_Na_ in human atrial cardiomyocytes under baseline conditions and in presence of 100 µM CBZ. CBZ significantly reduced I_Na_ density, without changes in V_1/2_ and k of activation (Figure 5D, bottom panels).

## 4 Discussion

The main findings of the present study are: 1) CBZ use is associated with increased SCA risk in the general population; 2) CBZ reduces cardiac AP upstroke velocity and I_Na_ in human and rabbit cardiomyocytes; 3) CBZ results in a tendency to (in rabbit) and significant (in human) cardiac AP shortening and reduces I_Ca,L_, while leaving sarcolemmal potassium currents unaltered. All of the observed effects are consistent with each other: reduction in cardiac AP upstroke velocity is well explained by reduction in I_Na_ (Berecki et al., 2010), and may, in turn, lead to reduction in cardiac excitability and conduction velocity of the excitation wavefront in the heart, as represented by CBZ-induced QRS interval prolongation (Leslie et al., 1983). It also facilitates reentrant excitation, VF/VT, and SCA, as shown for the use of class IC antiarrhythmic drugs (potent I_Na_ blockers) (Rogers et al., 1989), and in Brugada syndrome (where 20% of patients have an identifiable loss-of-function mutation in *SCN5A*, the gene that encodes the Na_V_1.5 α-subunit of the cardiac Na^+^ channel) (Meregalli et al., 2005). Previous case reports (Table 1) have reported findings that are consistent with these electrophysiological effects of CBZ. Accordingly, we found that CBZ use is associated with a 90% increase in the risk of SCA in the general population. These epidemiological findings are consistent with a previous study by Bardai et al. (2015) on the association of SCA with epilepsy and with the use of CBZ, which was conducted in a smaller patient set (10 cases used CBZ and 26 controls were included) and with less certain SCA ascertainment (no ECG documentation). In our study, we had no information regarding the epilepsy status. Hence, we could not adjust for epilepsy in the epidemiological analysis. This is an important limitation considering that epilepsy is associated with increased SCA risk (Surges et al., 2009). Therefore, our findings from the epidemiological analysis should be interpreted with caution. However, Bardai et al. (2015) found that the AEDs with putative cardiac I_Na_ blocking properties such as CBZ are similarly associated with an increased SCA risk. This was not only observed among patients with epilepsy, but also among patients who had no epilepsy (but used AEDs for other indications, e.g., neuralgia). Moreover, the observed association between CBZ and SCA remained unchanged after correction for epilepsy (Bardai et al., 2015). This suggested that the SCA risk associated with CBZ use resulted from the drug effect rather than from suffering epilepsy *per se*.

Of note, we measured the effects of different concentrations of CBZ (1–100 µM) *in vitro*, including concentrations corresponding to plasma levels that provide anticonvulsant effects (20–40 µM) (Bertilsson 1978). CBZ displays a high distribution volume, entering the bloodstream from tissue reserves (Charlier et al., 2021), which, together with the fine end-branches of the vasculature of the heart, would make sure that all cardiomyocytes (not only the cells on the surface) are exposed to the compounds in the blood and the extracellular fluid. Thus, the plasma CBZ concentration is a good measure of the concentration of free CBZ “seen” by the cardiomyocytes in the intact heart and in our *in vitro* experiments. Kennebäck et al. (1995) reported a CBZ plasma concentration of 26.1 ± 5.5 µM (mean ± SD) at a dose of 400 mg/day and 35.6 ± 5.9 μM at 800 mg/day in healthy volunteers. Correspondingly, our study showed that the CBZ-induced reduction of upstroke velocity was present at 10 μM at 2 Hz and faster, and at 30 μM at all pacing frequencies, which is thus within the range of therapeutic concentrations. Our observed reduction of upstroke velocity is consistent with findings in guinea-pig ventricular cardiomyocytes where 75 µM CBZ significantly reduced dV/dt_max_ at 1 Hz frequency stimulation by ≈ 13% (Delaunois et al., 2015).

We here compared our used CBZ concentrations to plasma concentrations in healthy volunteers. However, as reviewed by Bertilsson (1978), a poor correlation between the prescribed dose and the actual plasma concentration of CBZ is found in epileptic patients. Furthermore, CBZ plasma levels may be affected by several factors, among which age, pregnancy, and pharmacokinetic drug interactions, including interactions with both central nervous system and cardiovascular drugs (Bertilsson 1978; Panday et al., 2017). Consequently, CBZ plasma levels show a considerable inter-individual variability (Bertilsson 1978; Panday et al., 2017). On the one hand, plasma levels can be so low that therapeutic efficacy is lost, while on the other hand the therapeutic range of 4–10 or 4–12 μg/mL (17–42 or 17–51 μM, respectively) is exceeded in a substantial percentage of patients treated with CBZ (Shakya et al., 2008; Al-Balawi et al., 2020; Eroğlu et al., 2021; Grześk et al., 2021), which may have contributed to the observed cardiac arrhythmias of Table 1. Supratherapeutic CBZ plasma levels were found in 4.9% of their patients by Shakya et al. (2008), in 8.6% by Al-Balawi et al. (2020), in 16% by Eroğlu et al. (2021), and in 2.1% by Grześk et al. (2021).

The CBZ-induced changes in upstroke velocity support our epidemiological findings, and suggest that CBZ affects I_Na_ (Berecki et al., 2010). Indeed, we found that ≥30 µM CBZ reduced cardiac I_Na_ and that it affected various gating properties (hyperpolarizing shift in voltage dependency of inactivation and slower recovery from inactivation). Our finding is supported by previous studies on CBZ’s effects on cardiac and neuronal I_Na_ (Kuo et al., 1997; Sun et al., 2007; Sheets et al., 2008; Harmer et al., 2011; Theile and Cummins 2011). For example, Harmer et al. (2011) found an IC_50_ of 152 µM for Na_V_1.5 channels expressed in CHO cells, while IC_50_ values for “brain-type” Na^+^ channels expressed in HEK293 cells were 2.5 and 1.6 mM for Na_V_1.3 and Na_V_1.7 channels in resting state, respectively (Sheets et al., 2008). In resting state, tetrodotoxin-resistant (TTX-R) Na_V_1.8 channels had an IC_50_ of 840 µM in dorsal root ganglion cells (Sheets et al., 2008). CBZ-induced shift in voltage dependency of inactivation and slowed recovery of inactivation were also observed for Na_V_1.3, Na_V_1.7 and Na_V_1.8 channels (Sheets et al., 2008; Theile and Cummins 2011). This strengthens the notion that I_Na_ block is a plausible contributing mechanism of increased SCA risk associated with CBZ and likely other AEDs with similar cardiac electrophysiological effects. This notion may serve as a basis to adapt clinical procedures for prescription of CBZ with the aim of reducing SCA risk (Benassi et al., 1987). This may be achieved by identifying individuals who are vulnerable to this risk when prescription of I_Na_ blocking CBZ is considered. This may be based on identification of the clinical conditions that increase SCA risk in the context of I_Na_ block, similar to guidelines regarding the prescription of I_Na_ blocking (class IC) antiarrhythmic drugs in case of ischemic heart disease and heart failure (Greenberg et al., 1995). Also, procedures to screen for genetic vulnerability (pharmacogenetics) may be developed (Surges et al., 2009). Finally, at set out above, CBZ levels are affected by several factors and supratherapeutic CBZ levels have been found in a substantial percentage of CBZ users. Therefore, CBZ concentrations need to be closely evaluated (Panday et al., 2017; Charlier et al., 2021).

While I_Na_ block is a plausible mechanism underlying the higher SCA risk observed during CBZ use, there is less compelling evidence to support the notion that increased SCA risk results from changes in AP repolarization. We found mild effects of CBZ on AP repolarization as indicated by the tendency to (in rabbit cardiomyocytes) and significant (in human cardiomyocytes) APD_90_ shortening at 100 µM CBZ, which is above the reported plasma concentrations (Kennebäck et al., 1995). An AP shortening was also observed at 75 µM CBZ in guinea-pig ventricular myocytes at 1 Hz stimulation frequency (Delaunois et al., 2015), but QT intervals, ECG measures of the ventricular AP durations, were not affected by therapeutic doses of CBZ (Arhan et al., 2009; Amin et al., 2010; Dogan et al., 2010; Sathyaprabha et al., 2018). The mild extent of CBZ effects on AP repolarization fits with our voltage clamp experiments. We observed a lack of CBZ effects on the main cardiac repolarizing currents, I_K1_, I_K_ and I_to1_, consistent with previous findings in other tissues and expression systems (Wooltorton and Mathie 1993; Rundfeldt 1997; Kobayashi et al., 2009). CBZ (10–50 µM) had no effect on I_K_ in rat isolated sympathetic neurons (Wooltorton and Mathie 1993) and NG108-15 neuronal cells (Rundfeldt 1997), while it did not affect Kir2.1 currents (Kobayashi et al., 2009), with Kir2.1 as the major Kir isoform of I_K1_ channels in cardiac myocytes. Although one study reported that CBZ inhibited the I_Kr_ tail current, the CBZ dosages used in that study (250–500 µM) were much higher than recommended therapeutic concentrations (Danielsson et al., 2003). We found a mild reduction of the depolarizing current I_Ca,L_ at 100 µM. Although it agrees with findings in cultured rat hippocampus neurons (Ambrósio et al., 1999) and rat sensory spinal ganglion cells (Schirrmacher et al., 1995), it is unlikely that such a decrease contributes to the SCA increase and relates to CBZ-induced changes in whole heart parameters, because the reduction is rather small and only observed at 100 μM, which is above the therapeutic plasma concentrations (Kennebäck et al., 1995). It has been demonstrated that CBZ reduced connexin43 expression in cultured cardiomyocytes (Schirrmacher et al., 1995), but more studies are required to determine the exact role of cardiac connexins in the altered ECG parameters and arrhythmias by CBZ use, and our observation of increased SCA.

The effects of CBZ on APs and I_Na_ density of freshly isolated human atrial cardiomyocytes were only tested at 100 µM due to the limited availability of Ca^2+^-tolerant, non-depolarized cells (Verkerk et al., 2021). We used human atrial cardiomyocytes isolated from explanted hearts of patients (with various medications) with end-stage heart failure caused by ischemic cardiomyopathy. Although such cells may be in a diseased state, the main effects of CBZ on those human atrial cardiomyocytes were largely similar to those on ventricular cardiomyocytes of control rabbits, indicating that the effects of CBZ are also present in human conditions. The K^+^ currents and I_Ca,L_ were measured with very general voltage clamp protocols without specific solutions and/or blockers. Although such measurements might also involve small contributions of other membrane currents, the CBZ effects were assessed in paired experiments. In addition, our findings match with CBZ findings on membrane currents in non-cardiomyocytes (Wooltorton and Mathie 1993; Rundfeldt 1997; Ambrósio et al., 1999; Danielsson et al., 2003; Sheets et al., 2008; Kobayashi et al., 2009; Theile and Cummins 2011), indicating that the CBZ effects on these (net) currents were reliably characterized.

## 5 Conclusion

CBZ reduces cardiac depolarization by reducing I_Na_, and inducing an associated reduction of the AP upstroke velocity, in cardiomyocytes at therapeutic plasma concentrations. CBZ also affects cardiac repolarization, by reducing I_Ca,L_, and an associated reduction of AP duration, but only at relatively high concentrations. These electrophysiological effects may contribute to the found increased SCA risk upon CBZ use in the general population.

## Data Availability

The original contributions presented in the study are included in the article/Supplementary Material, further inquiries can be directed to the corresponding author.
