# Supplementary material for: Carbamazepine Increases the Risk of Sudden Cardiac Arrest by a Reduction of the Cardiac Sodium Current
Source: Front Cell Dev Biol. 2022 Jun 3;10:891996. doi: 10.3389/fcell.2022.891996 (PMC9204209; doi:10.3389/fcell.2022.891996)
Supplement: Supplementary file 1 [file DataSheet1.pdf]

Supplementary Material

1 Supplementary Methods and Materials

1.1 Isolation of Cardiomocytes

1.1.1 Rabbit Cardiomyocytes

Male New Zealand White rabbits (3.0–3.5 kg) were sedated and anaesthetized with 0.2 mL/kg Hypnorm® (0.32 mg/mL fentanyl-citrate and 10 mg/mL fluanisone; intramuscular; Janssen Pharmaceutics, Leiden, The Netherlands), heparinized (5000 IU heparin, LEO Pharma, Buckinghamshire, UK), and subsequently killed by injection of 1 mL/kg Nembutal® (60 mg/mL sodium pentobarbital; intravenous; Ceva Santé Animale B.V., Naaldwijk, The Netherlands). Hearts were excised, and transported to the laboratory in cold (4°C) Tyrode's solution containing (in mM): NaCl 128, KCl 4.7, $CaCl_2$ 1.5, $MgCl_2$ 0.6, $NaHCO_3$ 27, Na_2HPO_4 0.4, and glucose 11; pH 7.4 by equilibration with 95% O_2 and 5% CO_2. Subsequently, hearts were mounted on a Langendorff perfusion apparatus and left ventricular midmyocardial myocytes were isolated by enzymatic dissociation from the most apical part of the left midmyocardial ventricular free wall as described previously in detail (Verkerk et al., 2004).

1.1.2 Human Cardiomyocytes

Human atrial cardiomyocytes were isolated from explanted hearts of male patients with end-stage heart failure caused by ischemic cardiomyopathy. Directly after explantation, the hearts were transported to the laboratory in oxygenated modified Tyrode's solution containing (in mM): NaCl 140, KCl 5.4, $CaCl_2$ 1.8, $MgCl_2$ 1.0, glucose 5.5, HEPES 5.0; pH 7.4 (NaOH). Parts of both atria were cut into small cubic chunks (≈1 mm^3), and stored in modified Tyrode's solution (20°C) until the cell isolation procedure was started. To this end, atrial chunks were placed in nominally Ca^{2+}-free Tyrode's solution (20°C), i.e., modified Tyrode's solution without the $CaCl_2$, which was refreshed two times. Then, the chunks were incubated for 30–60 min in nominally Ca^{2+}-free Tyrode's solution (37°C) to which liberase III (0.22–0.25 U/mL; Roche Diagnostics, Mannheim, Germany), elastase (0.21–0.24 U/mL; Serva, Heidelberg, Germany), and pronase E (0.92 U/mL; Serva) were added. During the incubation period, the chunks were triturated through a pipette (tip diameter: 2.4 mm) and at regular intervals, the solution was microscopically examined for the

presence of dissociated cardiomyocytes. When single cells appeared, dissociation was stopped, and the chunks were transferred into a modified Kraft-Brühe (KB) solution (20°C), which was refreshed three times. KB was composed of (in mM): KCl 85, K_2HPO_4 30, $MgSO_4$ 5.0, glucose 20, pyruvic acid 5.0, creatine 5.0, taurine 30, β-hydroxybutyric acid 5.0, succinic acid 5.0, BSA 1%, Na_2ATP 2.0; pH 6.9 (KOH). Thereafter, single cells were obtained by trituration of the chunks (pipette tip diameter: 2 mm) for ≈2 min. Single cells were stored at room temperature in modified KB solution until use.

1.2 Patch Clamp Experiments

1.2.1 Recording Procedures

Action potentials (APs) and membrane currents were recorded in quiescent cardiomyocytes with smooth surfaces and clear striations with the ruptured-patch whole-cell configuration of the patch clamp technique using an Axopatch 200B amplifier (Molecular Devices, San Jose, CA, USA). Patch pipettes (borosilicate glass) were heat polished and had a resistance of 2–3 MΩ for recording APs, and Ca^{2+} and K^+ currents, while it was 1.4–1.8 MΩ for Na^+ current (I_{Na}) measurements. Voltage control, data acquisition, and analysis were accomplished using custom software (Ten Hoope et al., 2018). All signals were low-pass filtered (5 kHz) and digitized at 25 (APs), 10 (Ca^{2+} and K^+ currents), or 33 (I_{Na}) kHz. Cell membrane capacitance (C_m) was estimated as described previously (Verkerk et al., 2004), series resistance was compensated by ≥80%, and potentials were corrected for the estimated liquid junction potential (Barry and Lynch, 1991).

1.2.2 Membrane Current Analysis

Membrane currents were measured with specific voltage clamp protocols as depicted in the insets to Figures 3–5. For the L-type Ca^{2+} current ($I_{Ca,L}$), the delayed rectifier K^+ current (I_K), and the inward rectifier K^+ current (I_{K1}), we used a holding potential of −50 mV to inactivate I_{Na} and the transient outward K^+ current (I_{to1}), while I_{to1} and I_{Na} were measured from a holding potential of −80 and −120 mV, respectively. I_{Na} was measured with a double-pulse protocol (Figure 3A). During the first depolarizing pulses (P1), I_{Na} activates and the currents analyzed here are used to determine current-voltage (I-V) relationships and the voltage dependency of activation. The second pulse (P2) is used to determine the voltage dependency of inactivation. Recovery from inactivation was measured with a double-pulse protocol with two depolarizing steps (P1 and P2) from −120 to −20 mV and a variable interpulse interval (Figure 3E). Currents measured during P2 were normalized to currents measured during P1. I_{Na} protocols were applied once every 5 s. To determine the (in)activation characteristics of I_{Na}, current-voltage relationships were corrected for differences in

driving force and normalized to maximum peak current. Steady-state activation and inactivation curves were fit using the Boltzmann equation $I/I_{max} = A/\{1.0+\exp[(V_{1/2}-V)/k]\}$ to determine the membrane potential for half-maximal (in)activation $V_{1/2}$ and the slope factor k.

$I_{Ca,L}$, I_K, and I_{K1} were measured using 500-ms hyper- and depolarizing pulses (Figure 4A) once every 2 s. I_{to1} was measured using 500-ms depolarizing pulses applied every 5 s. A 5-ms prepulse to −40 mV (Figure 4G) served to activate and inactivate I_{Na}. We defined $I_{Ca,L}$ and I_{Na} as the difference between the peak inward current and the current at the end of a depolarizing voltage clamp step. I_{K1} was defined as the current at the end of hyperpolarizing voltage steps, whereas I_K was defined as the current at the end of depolarizing voltage steps (Figure 4, D and E). I_K in ventricular myocytes may consists of a slow and a rapid component (I_{Ks} and I_{Kr}, respectively) (Nerbonne and Kass, 2005). The presence of I_{Ks} in rabbit ventricular myocytes is debated (see Verkerk et al. (2011) and primary references cited therein). Previously, we were unable to detect I_{Ks}, while the I_{Kr} blocker E-4031 abolished tail current after 4-s depolarizing pulses from −50 to +40 mV completely (Verkerk et al., 2011). As we discussed previously (Verkerk et al., 2011), the absence of I_{Ks} in rabbit ventricular cardiomyocytes is likely related to differentially expressed I_{Ks} in rabbit ventricles with a markedly smaller I_{Ks} at the apex than at the base. In addition, I_{Ks} is much smaller in midmyocardial than in subepicardial and subendocardial myocytes. In our cell preparation method, we used midmyocardial myocytes from the apex of the heart. Therefore, I_K in our experiments is attributed to I_{Kr} rather than to I_{Ks}. I_{to1} was defined as the difference between peak transient outward current and the current at the end of the depolarizing voltage step.

1.2.3 Osmolarity

In previous studies (Van Borren et al., 2002; Verkerk et al., 2004), we have used a freezing-point depression-type osmometer (Knauer, Germany) and found that the measured and calculated osmolarity of solutions were indeed almost identical. The pH was set with ≈2.2 mM KOH, NaOH, or CsOH, which resulted in a calculated osmolarity of: (1) 314.1 mOsm for modified Tyrode's solution; (2) 320.4 mOsm for the pipette solution used in the AP, K^+ current, and Ca^{2+} current measurements; (3) 309.4 mOsm for the bath solution in the Na^+ current measurements; and (4) 307.4 mOsm for the pipette solution in the Na^+ current measurements.

References

Barry, P. H., and Lynch, J. W. (1991). Liquid junction potentials and small cell effects in patch-clamp analysis. *J. Membr. Biol.* 121, 101–117. doi: 10.1007/bf01870526

Nerbonne, J. M., and Kass, R. S. (2005). Molecular physiology of cardiac repolarization. *Physiol. Rev.* 85, 1205–1253. doi: 10.1152/physrev.00002.2005

Ten Hoope, W., Hollmann, M. W., De Bruin, K., Verberne, H. J., Verkerk, A. O., Tan, H. L., et al. (2018). Pharmacodynamics and pharmacokinetics of lidocaine in a rodent model of diabetic neuropathy. *Anesthesiology* 128, 609–619, doi: 10.1097/aln.0000000000002035

Van Borren, M. M. G. J., Verkerk, A. O., Vanharanta, S. K., Baartscheer, A., Coronel, R., and Ravesloot, J. H. (2002). Reduced swelling-activated Cl^- current densities in hypertrophied ventricular myocytes of rabbits with heart failure. *Cardiovasc. Res.* 53, 869–878. doi: 10.1016/s0008-6363(01)00507-7

Verkerk, A. O., Baartscheer, A., De Groot, J. R., Wilders, R., and Coronel, R. (2011). Etiology-dependency of ionic remodeling in cardiomyopathic rabbits. *Int. J. Cardiol.* 148, 154–160. doi: 10.1016/j.ijcard.2009.10.047

Verkerk, A. O., Tan, H. L., and Ravesloot, J. H. (2004). Ca^{2+}-activated Cl^- current reduces transmural electrical heterogeneity within the rabbit left ventricle. *Acta Physiol. Scand.* 180, 239–247. doi: 10.1111/j.0001-6772.2003.01252.x

Verkerk, A. O., Wilders, R., and Ravesloot, J. H. (2004). Identification of swelling-activated Cl^- current in rabbit cardiac Purkinje cells. *Cell. Mol. Life Sci.* 61, 1106–1113. doi: 10.1007/s00018-004-4028-9

2 Supplementary Table

Supplementary Table S1. Data of patients whose hearts were used for cellular electrophysiological studies

Patient	Age (yr)	Sex	Diagnosis	NYHA class	EF (%)	Clinical data	Previous medications
1	58	M	ICM	IV	19	—	antico, ACE, nit, diu, dig, Ca, dop
2	57	M	ICM	IV	15	VF (ICD)	antico, ACE, nit, diu, dop, β-block
3	61	M	ICM	IV	25	—	antico, ACE, nit, diu

Patient characteristics: NYHA class, New York Heart Association functional class; EF, left ventricular ejection fraction; ICM, ischemic cardiomyopathy; VF, ventricular fibrillation; ICD, implantable cardioverter-defibrillator. Previous medications: antico, anticoagulants; ACE, angiotensin-converting enzyme inhibitors; nit, nitrate; diu, diuretics; dig, digoxin; Ca, Ca-antagonists; dop, dopamine; β-block, β-blockers.

3 Supplementary Figures

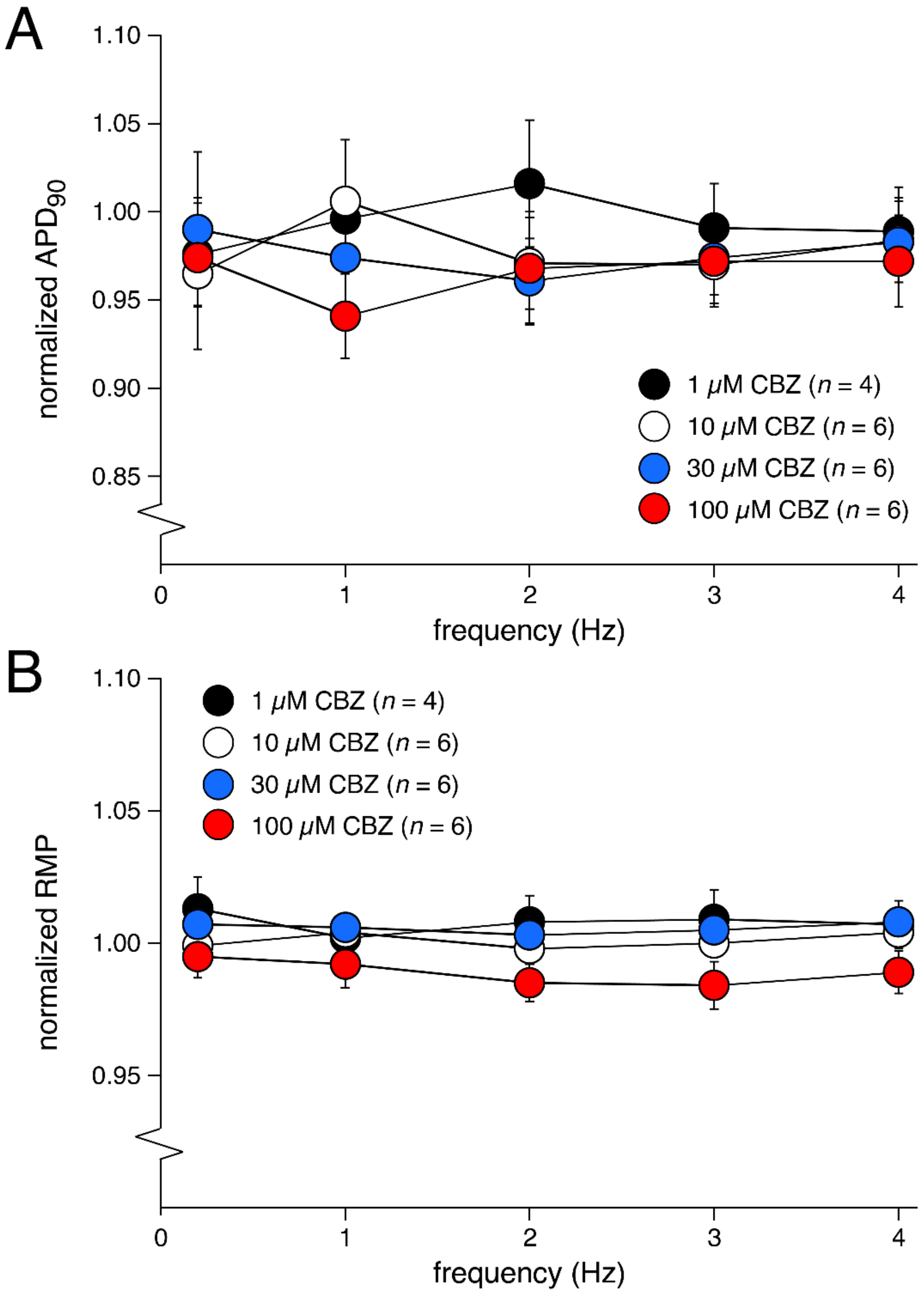

Supplementary Figure S1. (A) AP duration at 90% repolarization (APD_{90}) and **(B)** resting membrane potential (RMP) of rabbit ventricular cardiomyocytes at stimulus frequencies ranging from 0.2 to 4 Hz in presence of 1 to 100 µM carbamazepine (CBZ). APD_{90} and RMP values are normalized to baseline conditions. $P > 0.05$ at all stimulus frequencies and CBZ concentrations (Two-Way Repeated Measures ANOVA).

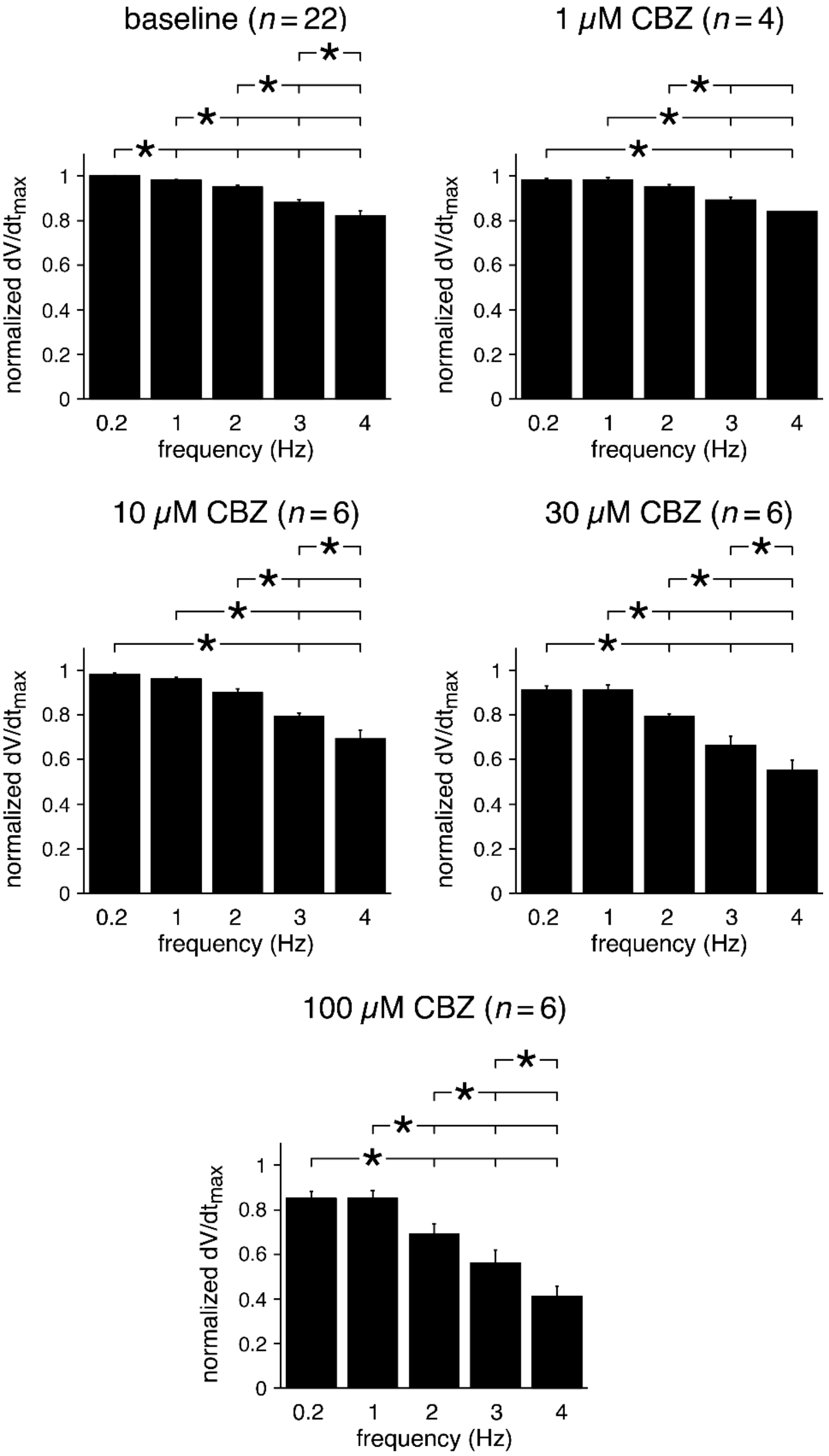

Supplementary Figure S2. Frequency dependency of the action potential upstroke velocity (dV/dt_{max}) of rabbit ventricular cardiomyocytes under baseline conditions and in presence of 1 to 100 µM carbamazepine (CBZ). Values are normalized to the highest dV/dt_{max} measured at stimulus frequencies ranging from 0.2 to 4 Hz under baseline conditions. *$P < 0.05$ (Two-Way Repeated Measures ANOVA).
